# Crystal structures and Hirshfeld surfaces of two 1,3-benzoxa­thiol-2-one derivatives

**DOI:** 10.1107/S2056989017018072

**Published:** 2018-01-01

**Authors:** Eliza de L. Chazin, Paola de S. Sanches, Thatyana R. A. Vasconcelos, Claudia R. B. Gomes, James L. Wardell, William T. A. Harrison

**Affiliations:** a Universidade Federal Fluminense, Instituto de Química, Programa de Pós-Graduação em Química, Rua Outeiro de São João Batista s/no, Centro, Niterói, 24020-141, RJ, Brazil; b Instituto de Tecnologia em Fármacos – Farmanguinhos, Fiocruz. R. Sizenando, Nabuco, 100, Manguinhos, 21041-250, Rio de Janeiro, RJ, Brazil; c Department of Chemistry, University of Aberdeen, Meston Walk, Aberdeen AB24 3UE, Scotland

**Keywords:** crystal structure, benzoxa­thiol-2-one, hydrogen bonds, Hirshfeld surface

## Abstract

The crystal structures of 6-meth­oxy-1,3-benzoxa­thiol-2-one, C_9_H_8_O_3_S, (I), and 2-oxo-1,3-benzoxa­thiol-6-yl acetate, C_9_H_6_O_4_S, (II), are described. Compound (I) is almost planar (r.m.s. deviation for the non-H atoms = 0.011 Å), whereas (II) shows a substantial twist between the fused-ring system and the acetate substituent [dihedral angle = 74.42 (3)°]. For both structures, the bond distances in the heterocyclic ring suggest that little if any conjugation occurs. In the crystal of (I), C—H⋯O hydrogen bonds link the mol­ecules into [1-11] chains incorporating alternating *R*_2_^2^(8) and *R*_2_^2^(12) inversion dimers. The extended structure of (II) features *C*(7) [201] chains linked by C—H⋯O hydrogen bonds, with further C—H⋯O bonds and weak π–π stacking inter­actions connecting the chains into a three-dimensional network. Hirshfeld fingerprint analyses for (I) and (II) are presented and discussed.

## Abstract 2

The packing motifs in the title compounds both feature C—H⋯O inter­actions but they show distinctly different Hirshfeld surface fingerprints.

## Chemical context   

1,3-Benzoxa­thiol-2-one and its derivatives have various biological properties including anti­bacterial, anti­mycotic, anti­oxidant, anti­tumor and anti-inflammatory activities (Vellasco Júnior *et al.*, 2011 ▸; Chazin *et al.*, 2015 ▸). They also act as inhibitors of carbonic anhydrase II (Barrese *et al.*, 2008 ▸) and mono­amine oxidase (Mostert *et al.*, 2016 ▸). The first synthesized 1,3-benzoxa­thiol-2-one, 6-hy­droxy-1,3-benzoxa­thiol-2-one C_7_H_4_O_3_S, also known as tioxolone or thioxolone, has been used for many years in the treatment of acne and other skin diseases (*e.g*. psoriasis) (Berg & Fiedler, 1959 ▸).

A recent study reported the syntheses and anti­fungal activities of some derivatives of tioxolone (Terra *et al.*, 2018 ▸). In the present article we report the crystal structures and Hirshfeld surface analyses of two compounds with different substituents at the 6-position of the ring system obtained in that study, *viz*. 6-meth­oxy-1,3-benzoxa­thiol-2-one, C_9_H_8_O_3_S, (I), and 2-oxo-1,3-benzoxa­thiol-6-yl acetate, C_9_H_8_O_3_S, (II).[Chem scheme1].
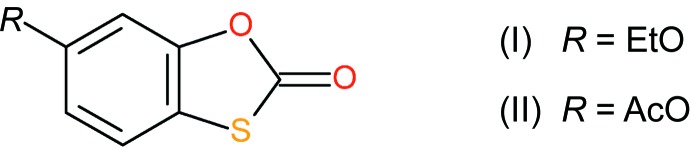


## Structural commentary   

**Figure 1:**
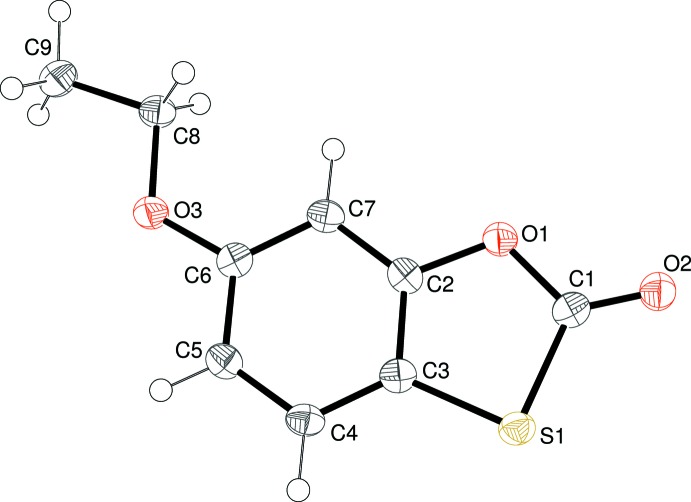
The mol­ecular structure of (I), showing 50% displacement ellipsoids.

Compound (I) crystallizes in space group *P* 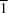 with one mol­ecule in the asymmetric unit (Fig. 1 ▸), which is almost planar (r.m.s. deviation for the non-hydrogen atoms = 0.011 Å): the eth­oxy side chain adopts an extended conformation [C6—O3—C8—C9 = 179.82 (12)°]. Within the oxa­thiol-2-one ring system, the C1—O1, C1=O2 and C1—S1 bond lengths are 1.3732 (18), 1.1905 (19) and 1.7766 (16)Å, respectively, and the O1—C1—S1 and C1—S1—C3 bond angles are 111.07 (11) and 90.62 (7)°, respectively. These distance data suggest that there is little if any conjugation (*i.e*. partial double-bond character) involving the C1—O1 and C1—S1 bonds with the C1=O2 group.

**Figure 2:**
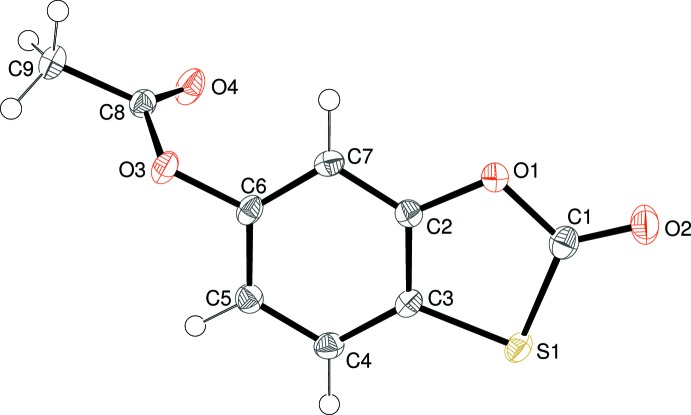
The mol­ecular structure of (II), showing 50% displacement ellipsoids.

A single mol­ecule of compound (II) makes up the asymmetric unit in space group *P*2_1_/*c* (Fig. 2 ▸). The ring system is almost planar (r.m.s. deviation for C1–C7/O1/S1 = 0.032 Å) but there is a substantial twist about the C6—O3 bond, as indicated by the dihedral angle between the ring system and the acetate group of 74.42 (3)°. Key geometrical data for the heterocyclic ring are C1—O1 = 1.3864 (15), C1=O2 = 1.1936 (15), C1—S1 = 1.7709 (13) Å, O1—C1—S1 = 111.57 (8) and C1—S1—C3 = 90.43 (6)°. These data are similar to the equivalent values for (I) and again indicate a lack of significant electronic delocalization within the oxa­thiol-2-one ring system.

## Supra­molecular features   

**Figure 3:**
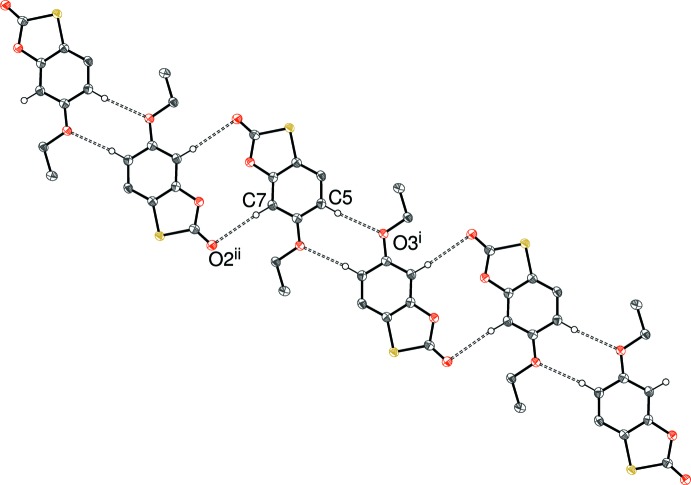
Fragment of a [1
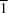
1] hydrogen-bonded chain in (I); all hydrogen atoms except H5 and H7 have been omitted for clarity. Symmetry codes as in Table 1 ▸.

In the crystal of (I), the mol­ecules are linked by C—H⋯O hydrogen bonds (Table 1 ▸). The C5—H5⋯O3^i^ [symmetry code: (i) −*x*, 1 − *y*, −*z*] link generates inversion dimers featuring 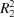
(8) loops. Based on its length, the C7—H7⋯O2^ii^ [symmetry code: (ii) 1 − *x*, −*y*, 1 − *z*] bond is much weaker, but if it is considered significant, it generates a second inversion dimer [with an 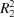
(12) graph-set symbol], which links the dimers into [1
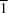
1] chains (Fig. 3 ▸). No C—H⋯π inter­actions could be identified in the crystal of (I) and any aromatic π–π stacking must be extremely weak, as the shortest centroid–centroid separation is 3.9149 (10) Å.

**Figure 4:**
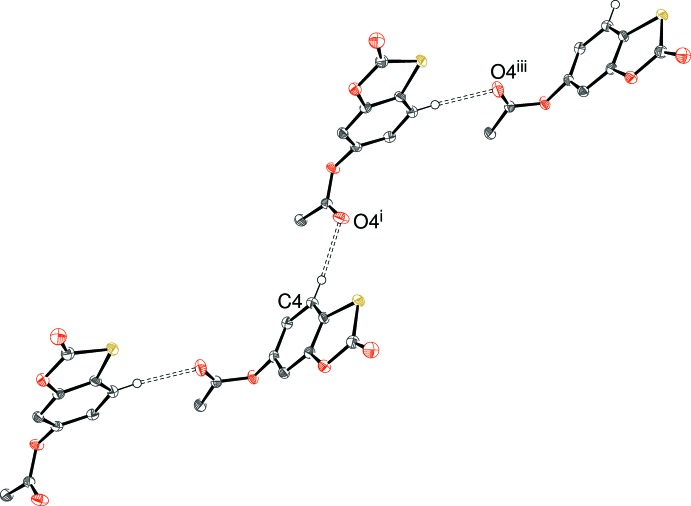
Fragment of a [201] hydrogen-bonded chain in (II); all hydrogen atoms except H4 omitted for clarity. Symmetry codes as in Table 2 ▸; additionally (iii) *x* − 2, *y*, *z* − 1.

In the crystal of (II), C4—H4⋯O4^i^ [symmetry code: (i) *x* − 1, 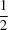 − *y*, *z* − 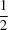] hydrogen bonds (Table 2 ▸) link the mol­ecules into *C*(7) chains propagating in [201], with adjacent mol­ecules in the chain related by *c*-glide symmetry (plus translation) (Fig. 4 ▸). The C9—H9*C*⋯O2^ii^ [symmetry code: (ii) 1 − *x*, −*y*, 1 − *z*] bonds arising from the methyl group generate inversion dimers [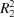
(16) loops], which connect the chains into a three-dimensional network. There are no C—H⋯π bonds in the crystal of (II) but the packing is consolidated by weak aromatic π–π stacking between inversion-related C2–C7 benzene rings with a centroid–centroid separation of 3.7220 (8) Å.

## Hirshfeld analyses   

**Figure 5:**
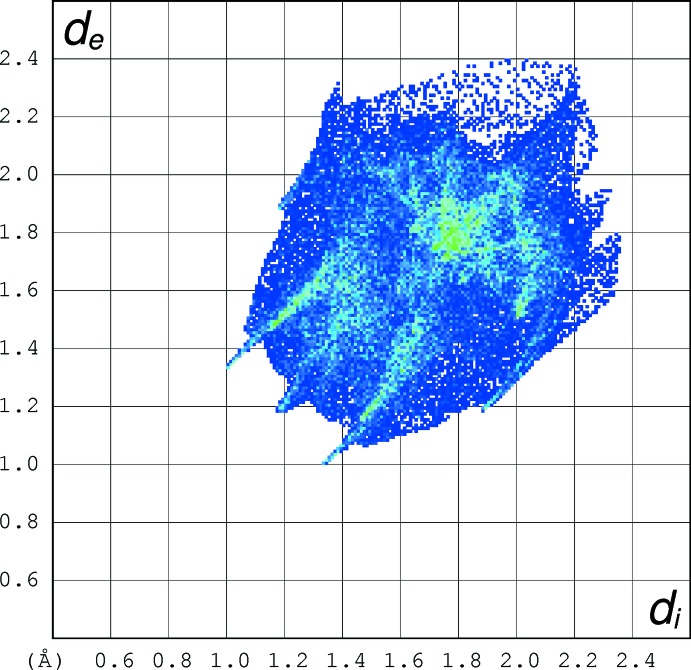
Hirshfeld fingerprint plot for (I)

**Figure 6:**
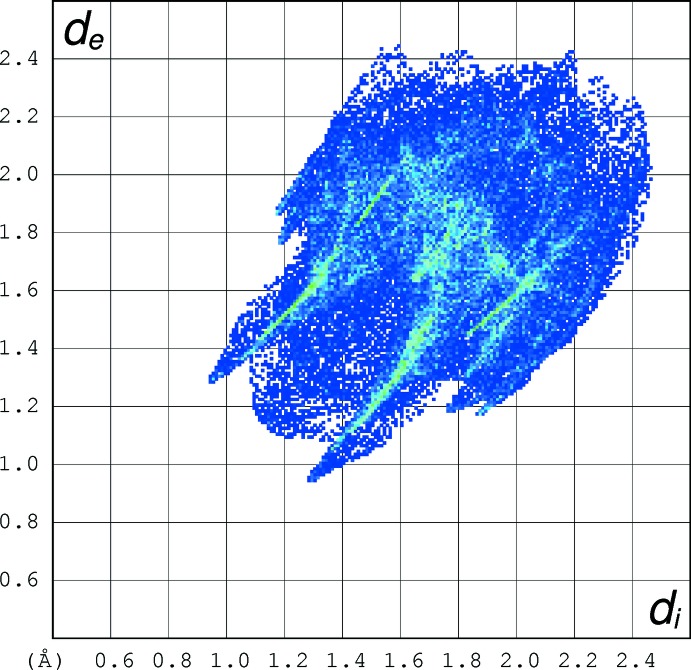
Hirshfeld fingerprint plot for (II)

**Figure 7:**
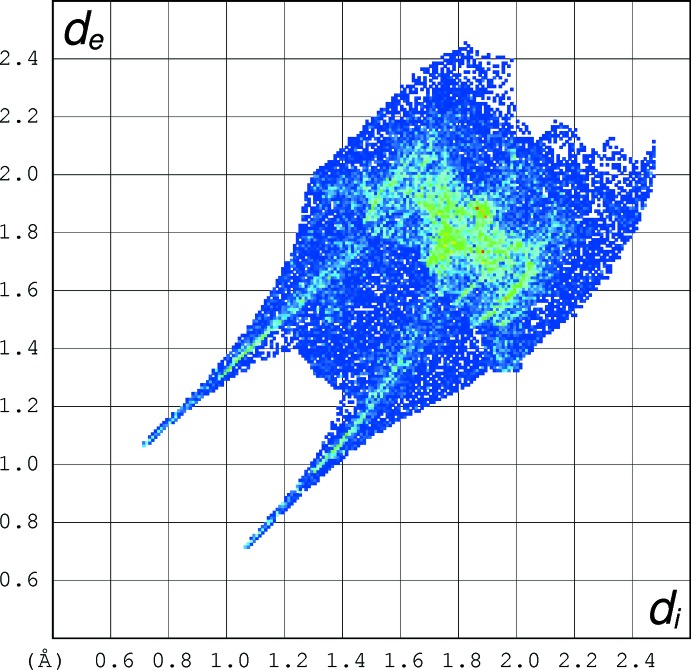
Hirshfeld fingerprint plot for tioxolone [atomic coordinates from Byres & Cox (2004 ▸)].

Hirshfeld surface fingerprint plots for (I) (Fig. 5 ▸), (II) (Fig. 6 ▸) and tioxolone (refcode: EVOQEL), which features classical O—H⋯O hydrogen bonds (Byres & Cox, 2004 ▸) (Fig. 7 ▸) were calculated with *CrystalExplorer17* (Turner *et al.*, 2017 ▸). The plot for EVOQEL has very pronounced ‘wingtip’ features that correspond to the short, classical O—H⋯O hydrogen bond found in this structure (compare: McKinnon *et al.*, 2007 ▸). In (I) and (II), the wingtips associated with the longer and presumably weaker C—H⋯O bonds are far less pronounced.

**Figure 8:**
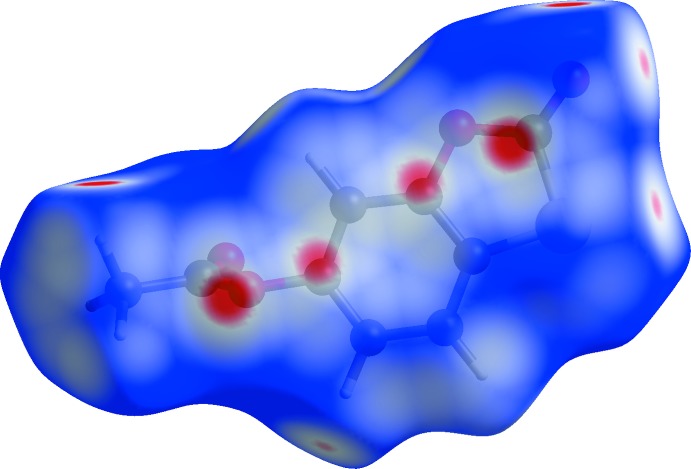
Hirshfeld surface plot mapped over *d*_norm_ for (II) showing red spots associated with short C⋯C and C⋯O contacts: the slightly smaller spots refer to the former.

When the fingerprint plots are decomposed into the separate types of contacts (McKinnon *et al.*, 2007 ▸), some inter­esting differences arise (Table 3 ▸): as a percentage of surface inter­actions, H⋯H contacts (*i.e*. van der Waals inter­actions) are far more prominent in (I) than in (II), which is comparable with EVOQEL, whereas C⋯H/H⋯C contacts are similar for the three structures. The O⋯H/H⋯O contacts are the most important contributors in all three structures, and in (II) they actually contribute a higher percentage to the surface than in EVOQEL, despite the fact that EVOQEL features both O—H⋯O and C—H⋯O hydrogen bonds and only one of its hydrogen atoms is not involved in such bonds (Byres & Cox, 2004 ▸). The C⋯C contacts (associated with aromatic π–π stacking) contribute a small percentage in (I) and (II) and about twice the amount in EVOQEL where the shortest centroid–centroid separation is 3.508 (2) Å. Despite the small percentage for (II), the Hirshfeld surface (Fig. 8 ▸) clearly shows red spots associated with these contacts. The S⋯H/H⋯S contacts are similar in the three structures, and not insignificant at ∼10% of the surfaces, but they can hardly represent directional C—H⋯S hydrogen bonds, as the shortest H⋯S separations (3.21, 3.11 and 3.28 Å in (I), (II) and EVOQEL, respectively) are much longer than the van der Waals contact distance (Bondi, 1964 ▸) of 3.00 Å for H and S. Finally, S⋯O/O⋯S contacts have very different contributions in the three structures: negligible in (I), but clearly present in (II) and EVOQEL. This seems to correlate with the shortest S⋯O contact distances of 3.623 (2), 3.2742 (10) and 3.341 (2) Å for (I), (II) and EVOQEL, respectively: the distance in (II) is actually slightly shorter than the van der Waals contact distance of 3.32Å for sulfur and oxygen,

## Database survey   

A survey of of the Cambridge Structural Database (Groom *et al.*, 2016 ▸: updated to September 2017) for the benzoxa­thiol-2-one fused ring system (any substituents) yielded just three matches, *viz*. 6-hy­droxy-1,3-benzoxa­thiol-2-one (refcode: EVOQEL; Byres & Cox, 2004 ▸); *N*-(4,7-dimethyl-2-oxo-benzo[1,3]oxa­thiol-5-yl)-4-benzene­sulfonamide (NAJQOG; Avdeenko *et al.*, 2009 ▸); 7-phenyl-1,3-benzoxa­thiol-2-one (JOSGUV; Zhao *et al.*, 2014 ▸). To this list may be added the structure of 6-meth­oxy-5-nitro­benzo[*d*][1,3]oxa­thiol-2-one (CCDC deposition number 1404755) as recently reported by Terra *et al.* (2017 ▸).

## Synthesis and crystallization   

Compounds (I) and (II) were prepared as described previously (Terra *et al.*, 2018 ▸) and recrystallized from methanol solution as colourless plates of (I) and colourless blocks of (II).

## Refinement   

Crystal data, data collection and structure refinement details are summarized in Table 4 ▸. The hydrogen atoms were geometrically placed (C—H = 0.95–0.99 Å) and refined as riding atoms. The constraint *U*_iso_(H) = 1.2*U*_eq_(carrier) or 1.5*U*_eq_(methyl carrier) was applied in all cases. The methyl groups were allowed to rotate, but not to tip, to best fit the electron density.

## Supplementary Material

Crystal structure: contains datablock(s) I, II, global. DOI: 10.1107/S2056989017018072/nk2242sup1.cif

Structure factors: contains datablock(s) I. DOI: 10.1107/S2056989017018072/nk2242Isup2.hkl

Supporting information file. DOI: 10.1107/S2056989017018072/nk2242Isup4.cml

Structure factors: contains datablock(s) II. DOI: 10.1107/S2056989017018072/nk2242IIsup3.hkl

Supporting information file. DOI: 10.1107/S2056989017018072/nk2242IIsup5.cml

CCDC references: 1404758, 1404756

Additional supporting information:  crystallographic information; 3D view; checkCIF report

## Figures and Tables

**Table 1:** Hydrogen-bond geometry (Å, °) for (I)

*D*—H⋯*A*	*D*—H	H⋯*A*	*D*⋯*A*	*D*—H⋯*A*
C5—H5⋯O3^i^	0.95	2.48	3.432 (2)	175
C7—H7⋯O2^ii^	0.95	2.66	3.5983 (19)	170

Symmetry codes: (i) 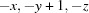; (ii) 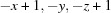.

**Table 2:** Hydrogen-bond geometry (Å, °) for (II)

*D*—H⋯*A*	*D*—H	H⋯*A*	*D*⋯*A*	*D*—H⋯*A*
C4—H4⋯O4^i^	0.95	2.35	3.2606 (15)	159
C9—H9*C*⋯O2^ii^	0.98	2.50	3.4030 (16)	153

Symmetry codes: (i) 
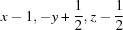
; (ii) 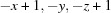.

**Table 3:** Hirshfeld contact inter­actions (%)

Contact type	(I)	(II)	EVOQEL
H⋯H	28.7	14.8	13.5
O⋯H/H⋯O	30.1	39.5	36.0
C⋯H/H⋯C	11.3	13.4	8.6
S⋯H/H⋯S	11.1	10.8	8.8
C⋯C	5.9	4.4	10.4
S⋯O/O⋯S	1.5	7.0	10.1

**Table 4:** Experimental details

	(I)	(II)
Crystal data		
Chemical formula	C_9_H_8_O_3_S	C_9_H_6_O_4_S
*M*_r_	196.21	210.20
Crystal system, space group	Triclinic, *P* 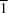	Monoclinic, *P*2_1_/*c*
Temperature (K)	100	100
*a*, *b*, *c* (Å)	3.9149 (6), 10.3237 (12), 11.7003 (14)	5.6232 (5), 14.5650 (14), 10.8572 (11)
α, β, γ (°)	66.526 (6), 81.110 (9), 84.349 (9)	90, 96.045 (2), 90
*V* (Å^3^)	428.18 (10)	884.28 (15)
*Z*	2	4
Radiation type	Mo *K*α	Mo *K*α
μ (mm^−1^)	0.35	0.35
Crystal size (mm)	0.17 × 0.17 × 0.03	0.26 × 0.11 × 0.08

Data collection		
Diffractometer	Rigaku Saturn CCD	Rigaku Saturn CCD
Absorption correction	Multi-scan (*FS_ABSCOR*; Rigaku, 2013 ▸)	Multi-scan (*FS_ABSCOR*; Rigaku, 2013 ▸)
*T*_min_, *T*_max_	0.714, 1.000	0.829, 1.000
No. of measured, independent and observed [*I* > 2σ(*I*)] reflections	5189, 1674, 1540	11487, 2028, 1903
*R*_int_	0.040	0.036
(sin θ/λ)_max_ (Å^−1^)	0.617	0.650

Refinement		
*R*[*F* ^2^ > 2σ(*F* ^2^)], *wR*(*F* ^2^), *S*	0.034, 0.094, 1.04	0.028, 0.081, 1.10
No. of reflections	1674	2028
No. of parameters	119	128
H-atom treatment	H-atom parameters constrained	H-atom parameters constrained
Δρ_max_, Δρ_min_ (e Å^−3^)	0.52, −0.20	0.33, −0.21

Computer programs: *CrystalClear* (Rigaku, 2014 ▸), *SHELXS97* (Sheldrick, 2008 ▸), *SHELXL2014* (Sheldrick, 2015 ▸), *ORTEP-3 for Windows* (Farrugia, 2012 ▸) and *publCIF* (Westrip, 2010 ▸).

## supplementary crystallographic information

### 6-Methoxy-1,3-benzoxathiol-2-one (I) Crystal data

**Table:**

C_9_H_8_O_3_S	*Z* = 2
*M**_r_* = 196.21	*F*(000) = 204
Triclinic, *P*1	*D*_x_ = 1.522 Mg m^−^^3^
*a* = 3.9149 (6) Å	Mo *K*α radiation, λ = 0.71073 Å
*b* = 10.3237 (12) Å	Cell parameters from 1390 reflections
*c* = 11.7003 (14) Å	θ = 1.9–27.5°
α = 66.526 (6)°	µ = 0.35 mm^−^^1^
β = 81.110 (9)°	*T* = 100 K
γ = 84.349 (9)°	Plate, colourless
*V* = 428.18 (10) Å^3^	0.17 × 0.17 × 0.03 mm

### 6-Methoxy-1,3-benzoxathiol-2-one (I) Data collection

**Table:**

Rigaku Saturn CCD diffractometer	1540 reflections with *I* > 2σ(*I*)
ω scans	*R*_int_ = 0.040
Absorption correction: multi-scan (*FS_ABSCOR*; Rigaku, 2013)	θ_max_ = 26.0°, θ_min_ = 1.9°
*T*_min_ = 0.714, *T*_max_ = 1.000	*h* = −4→4
5189 measured reflections	*k* = −12→12
1674 independent reflections	*l* = −14→14

### 6-Methoxy-1,3-benzoxathiol-2-one (I) Refinement

**Table:**

Refinement on *F*^2^	Primary atom site location: structure-invariant direct methods
Least-squares matrix: full	Hydrogen site location: inferred from neighbouring sites
*R*[*F*^2^ > 2σ(*F*^2^)] = 0.034	H-atom parameters constrained
*wR*(*F*^2^) = 0.094	*w* = 1/[σ^2^(*F*_o_^2^) + (0.0607*P*)^2^ + 0.0766*P*] where *P* = (*F*_o_^2^ + 2*F*_c_^2^)/3
*S* = 1.04	(Δ/σ)_max_ < 0.001
1674 reflections	Δρ_max_ = 0.52 e Å^−^^3^
119 parameters	Δρ_min_ = −0.20 e Å^−^^3^
0 restraints	

### 6-Methoxy-1,3-benzoxathiol-2-one (I) Special details

**Table:**

Geometry. All esds (except the esd in the dihedral angle between two l.s. planes) are estimated using the full covariance matrix. The cell esds are taken into account individually in the estimation of esds in distances, angles and torsion angles; correlations between esds in cell parameters are only used when they are defined by crystal symmetry. An approximate (isotropic) treatment of cell esds is used for estimating esds involving l.s. planes.

### 6-Methoxy-1,3-benzoxathiol-2-one (I) Fractional atomic coordinates and isotropic or equivalent isotropic displacement parameters (Å^2^)

**Table:**

	*x*	*y*	*z*	*U*_iso_*/*U*_eq_	
C1	0.0898 (4)	0.15148 (16)	0.62375 (14)	0.0238 (3)	
C2	0.1487 (4)	0.21490 (16)	0.40934 (14)	0.0221 (3)	
C3	−0.0473 (4)	0.33140 (16)	0.41485 (14)	0.0221 (3)	
C4	−0.1375 (4)	0.43830 (16)	0.30435 (15)	0.0238 (3)	
H4	−0.2733	0.5190	0.3064	0.029*	
C5	−0.0252 (4)	0.42431 (16)	0.19181 (15)	0.0245 (3)	
H5	−0.0854	0.4961	0.1157	0.029*	
C6	0.1770 (4)	0.30535 (16)	0.18846 (14)	0.0223 (3)	
C7	0.2673 (4)	0.19732 (16)	0.29857 (14)	0.0230 (3)	
H7	0.4028	0.1161	0.2975	0.028*	
C8	0.4817 (4)	0.18554 (16)	0.06172 (15)	0.0240 (3)	
H8A	0.3576	0.0970	0.1095	0.029*	
H8B	0.7026	0.1773	0.0959	0.029*	
C9	0.5494 (4)	0.21106 (18)	−0.07580 (15)	0.0283 (4)	
H9A	0.6977	0.1336	−0.0867	0.042*	
H9B	0.6651	0.3005	−0.1224	0.042*	
H9C	0.3294	0.2157	−0.1077	0.042*	
O1	0.2250 (3)	0.11453 (11)	0.52491 (10)	0.0244 (3)	
O2	0.1322 (3)	0.07887 (11)	0.72936 (10)	0.0282 (3)	
O3	0.2727 (3)	0.30495 (11)	0.07165 (10)	0.0251 (3)	
S1	−0.14241 (9)	0.31705 (4)	0.57093 (3)	0.02391 (16)	

### 6-Methoxy-1,3-benzoxathiol-2-one (I) Atomic displacement parameters (Å^2^)

**Table:**

	*U*^11^	*U*^22^	*U*^33^	*U*^12^	*U*^13^	*U*^23^
C1	0.0236 (8)	0.0232 (7)	0.0273 (8)	−0.0042 (6)	−0.0017 (6)	−0.0124 (6)
C2	0.0220 (7)	0.0193 (7)	0.0254 (8)	−0.0039 (6)	−0.0056 (6)	−0.0076 (6)
C3	0.0218 (7)	0.0212 (7)	0.0264 (8)	−0.0034 (6)	−0.0037 (6)	−0.0117 (6)
C4	0.0228 (7)	0.0213 (8)	0.0301 (8)	0.0010 (6)	−0.0056 (6)	−0.0125 (6)
C5	0.0257 (8)	0.0216 (7)	0.0273 (8)	−0.0009 (6)	−0.0069 (6)	−0.0096 (6)
C6	0.0214 (7)	0.0222 (8)	0.0258 (7)	−0.0045 (6)	−0.0026 (6)	−0.0112 (6)
C7	0.0223 (7)	0.0201 (7)	0.0286 (8)	−0.0013 (6)	−0.0042 (6)	−0.0110 (6)
C8	0.0245 (7)	0.0216 (7)	0.0280 (8)	0.0002 (6)	−0.0040 (6)	−0.0120 (6)
C9	0.0289 (8)	0.0302 (8)	0.0288 (8)	−0.0026 (7)	−0.0007 (6)	−0.0153 (7)
O1	0.0290 (6)	0.0210 (5)	0.0239 (6)	0.0013 (4)	−0.0044 (4)	−0.0096 (4)
O2	0.0335 (6)	0.0260 (6)	0.0246 (6)	−0.0001 (5)	−0.0037 (5)	−0.0095 (5)
O3	0.0300 (6)	0.0221 (6)	0.0247 (6)	0.0026 (4)	−0.0044 (4)	−0.0112 (5)
S1	0.0252 (2)	0.0234 (2)	0.0259 (2)	0.00092 (16)	−0.00403 (16)	−0.01255 (17)

### 6-Methoxy-1,3-benzoxathiol-2-one (I) Geometric parameters (Å, º)

**Table:**

C1—O2	1.1905 (19)	C5—H5	0.9500
C1—O1	1.3732 (18)	C6—O3	1.3617 (18)
C1—S1	1.7766 (16)	C6—C7	1.395 (2)
C2—C3	1.380 (2)	C7—H7	0.9500
C2—C7	1.383 (2)	C8—O3	1.4452 (18)
C2—O1	1.3926 (18)	C8—C9	1.508 (2)
C3—C4	1.394 (2)	C8—H8A	0.9900
C3—S1	1.7552 (16)	C8—H8B	0.9900
C4—C5	1.381 (2)	C9—H9A	0.9800
C4—H4	0.9500	C9—H9B	0.9800
C5—C6	1.405 (2)	C9—H9C	0.9800
			
O2—C1—O1	122.22 (14)	C2—C7—C6	116.34 (14)
O2—C1—S1	126.71 (12)	C2—C7—H7	121.8
O1—C1—S1	111.07 (11)	C6—C7—H7	121.8
C3—C2—C7	123.62 (14)	O3—C8—C9	107.02 (12)
C3—C2—O1	114.96 (13)	O3—C8—H8A	110.3
C7—C2—O1	121.41 (13)	C9—C8—H8A	110.3
C2—C3—C4	119.56 (14)	O3—C8—H8B	110.3
C2—C3—S1	110.37 (11)	C9—C8—H8B	110.3
C4—C3—S1	130.07 (12)	H8A—C8—H8B	108.6
C5—C4—C3	118.55 (14)	C8—C9—H9A	109.5
C5—C4—H4	120.7	C8—C9—H9B	109.5
C3—C4—H4	120.7	H9A—C9—H9B	109.5
C4—C5—C6	120.85 (14)	C8—C9—H9C	109.5
C4—C5—H5	119.6	H9A—C9—H9C	109.5
C6—C5—H5	119.6	H9B—C9—H9C	109.5
O3—C6—C7	123.91 (13)	C1—O1—C2	112.97 (12)
O3—C6—C5	115.01 (13)	C6—O3—C8	117.75 (12)
C7—C6—C5	121.08 (14)	C3—S1—C1	90.62 (7)
			
C7—C2—C3—C4	0.7 (2)	C5—C6—C7—C2	−0.4 (2)
O1—C2—C3—C4	179.88 (13)	O2—C1—O1—C2	−179.25 (14)
C7—C2—C3—S1	−179.08 (11)	S1—C1—O1—C2	0.23 (15)
O1—C2—C3—S1	0.14 (16)	C3—C2—O1—C1	−0.24 (18)
C2—C3—C4—C5	−0.3 (2)	C7—C2—O1—C1	178.99 (12)
S1—C3—C4—C5	179.33 (12)	C7—C6—O3—C8	−0.3 (2)
C3—C4—C5—C6	−0.3 (2)	C5—C6—O3—C8	179.90 (13)
C4—C5—C6—O3	−179.56 (13)	C9—C8—O3—C6	179.82 (12)
C4—C5—C6—C7	0.6 (2)	C2—C3—S1—C1	−0.01 (11)
C3—C2—C7—C6	−0.3 (2)	C4—C3—S1—C1	−179.71 (15)
O1—C2—C7—C6	−179.47 (13)	O2—C1—S1—C3	179.32 (15)
O3—C6—C7—C2	179.87 (13)	O1—C1—S1—C3	−0.13 (11)

### 6-Methoxy-1,3-benzoxathiol-2-one (I) Hydrogen-bond geometry (Å, º)

**Table:**

*D*—H···*A*	*D*—H	H···*A*	*D*···*A*	*D*—H···*A*
C5—H5···O3^i^	0.95	2.48	3.432 (2)	175
C7—H7···O2^ii^	0.95	2.66	3.5983 (19)	170

Symmetry codes: (i) −*x*, −*y*+1, −*z*; (ii) −*x*+1, −*y*, −*z*+1.

### 2-Oxo-1,3-benzoxathiol-6-yl acetate (II) Crystal data

**Table:**

C_9_H_6_O_4_S	*F*(000) = 432
*M**_r_* = 210.20	*D*_x_ = 1.579 Mg m^−^^3^
Monoclinic, *P*2_1_/*c*	Mo *K*α radiation, λ = 0.71073 Å
*a* = 5.6232 (5) Å	Cell parameters from 3355 reflections
*b* = 14.5650 (14) Å	θ = 2.3–27.5°
*c* = 10.8572 (11) Å	µ = 0.35 mm^−^^1^
β = 96.045 (2)°	*T* = 100 K
*V* = 884.28 (15) Å^3^	Block, colourless
*Z* = 4	0.26 × 0.11 × 0.08 mm

### 2-Oxo-1,3-benzoxathiol-6-yl acetate (II) Data collection

**Table:**

Rigaku Saturn CCD diffractometer	1903 reflections with *I* > 2σ(*I*)
ω scans	*R*_int_ = 0.036
Absorption correction: multi-scan (*FS_ABSCOR*; Rigaku, 2013)	θ_max_ = 27.5°, θ_min_ = 2.4°
*T*_min_ = 0.829, *T*_max_ = 1.000	*h* = −7→7
11487 measured reflections	*k* = −18→18
2028 independent reflections	*l* = −14→14

### 2-Oxo-1,3-benzoxathiol-6-yl acetate (II) Refinement

**Table:**

Refinement on *F*^2^	Primary atom site location: structure-invariant direct methods
Least-squares matrix: full	Hydrogen site location: inferred from neighbouring sites
*R*[*F*^2^ > 2σ(*F*^2^)] = 0.028	H-atom parameters constrained
*wR*(*F*^2^) = 0.081	*w* = 1/[σ^2^(*F*_o_^2^) + (0.0423*P*)^2^ + 0.328*P*] where *P* = (*F*_o_^2^ + 2*F*_c_^2^)/3
*S* = 1.10	(Δ/σ)_max_ = 0.001
2028 reflections	Δρ_max_ = 0.33 e Å^−^^3^
128 parameters	Δρ_min_ = −0.21 e Å^−^^3^
0 restraints	

### 2-Oxo-1,3-benzoxathiol-6-yl acetate (II) Special details

**Table:**

Geometry. All esds (except the esd in the dihedral angle between two l.s. planes) are estimated using the full covariance matrix. The cell esds are taken into account individually in the estimation of esds in distances, angles and torsion angles; correlations between esds in cell parameters are only used when they are defined by crystal symmetry. An approximate (isotropic) treatment of cell esds is used for estimating esds involving l.s. planes.

### 2-Oxo-1,3-benzoxathiol-6-yl acetate (II) Fractional atomic coordinates and isotropic or equivalent isotropic displacement parameters (Å^2^)

**Table:**

	*x*	*y*	*z*	*U*_iso_*/*U*_eq_	
C1	0.1830 (2)	0.03032 (8)	0.18546 (11)	0.0210 (2)	
C2	0.1514 (2)	0.07946 (8)	0.38446 (11)	0.0167 (2)	
C3	−0.0602 (2)	0.11624 (8)	0.32717 (11)	0.0174 (2)	
C4	−0.2186 (2)	0.16130 (8)	0.39666 (11)	0.0194 (2)	
H4	−0.3625	0.1872	0.3580	0.023*	
C5	−0.1609 (2)	0.16746 (8)	0.52441 (11)	0.0191 (2)	
H5	−0.2657	0.1979	0.5742	0.023*	
C6	0.0505 (2)	0.12894 (8)	0.57848 (10)	0.0175 (2)	
C7	0.2139 (2)	0.08489 (8)	0.51080 (11)	0.0175 (2)	
H7	0.3595	0.0601	0.5491	0.021*	
C8	0.2608 (2)	0.18164 (8)	0.76657 (11)	0.0183 (2)	
C9	0.2792 (2)	0.16577 (9)	0.90343 (11)	0.0222 (3)	
H9A	0.1197	0.1535	0.9284	0.033*	
H9B	0.3465	0.2204	0.9467	0.033*	
H9C	0.3833	0.1129	0.9248	0.033*	
O1	0.29007 (15)	0.03350 (6)	0.30638 (8)	0.01950 (19)	
O2	0.27313 (17)	−0.00958 (7)	0.10631 (8)	0.0282 (2)	
O3	0.08651 (15)	0.12684 (6)	0.70829 (7)	0.0207 (2)	
O4	0.37694 (17)	0.23332 (7)	0.71088 (8)	0.0275 (2)	
S1	−0.09135 (5)	0.09103 (2)	0.16863 (3)	0.02017 (11)	

### 2-Oxo-1,3-benzoxathiol-6-yl acetate (II) Atomic displacement parameters (Å^2^)

**Table:**

	*U*^11^	*U*^22^	*U*^33^	*U*^12^	*U*^13^	*U*^23^
C1	0.0246 (6)	0.0209 (6)	0.0177 (6)	−0.0017 (5)	0.0030 (4)	0.0015 (4)
C2	0.0164 (5)	0.0158 (5)	0.0181 (6)	−0.0015 (4)	0.0026 (4)	−0.0003 (4)
C3	0.0194 (5)	0.0177 (5)	0.0148 (5)	−0.0023 (4)	−0.0004 (4)	0.0001 (4)
C4	0.0180 (5)	0.0194 (6)	0.0202 (6)	0.0019 (4)	−0.0009 (4)	0.0003 (4)
C5	0.0194 (5)	0.0185 (6)	0.0196 (6)	−0.0010 (4)	0.0028 (4)	−0.0020 (4)
C6	0.0208 (5)	0.0178 (5)	0.0137 (5)	−0.0055 (4)	0.0007 (4)	0.0004 (4)
C7	0.0158 (5)	0.0182 (6)	0.0178 (6)	−0.0017 (4)	−0.0008 (4)	0.0019 (4)
C8	0.0201 (5)	0.0182 (5)	0.0163 (5)	0.0003 (4)	0.0005 (4)	−0.0020 (4)
C9	0.0287 (6)	0.0235 (6)	0.0140 (5)	−0.0009 (5)	0.0007 (4)	−0.0007 (4)
O1	0.0190 (4)	0.0230 (4)	0.0167 (4)	0.0017 (3)	0.0025 (3)	−0.0010 (3)
O2	0.0336 (5)	0.0321 (5)	0.0200 (5)	0.0048 (4)	0.0076 (4)	−0.0030 (4)
O3	0.0239 (4)	0.0251 (5)	0.0128 (4)	−0.0076 (3)	0.0006 (3)	0.0003 (3)
O4	0.0317 (5)	0.0327 (5)	0.0176 (4)	−0.0140 (4)	0.0008 (4)	−0.0002 (4)
S1	0.02389 (18)	0.02219 (18)	0.01374 (17)	0.00119 (11)	−0.00123 (12)	0.00007 (10)

### 2-Oxo-1,3-benzoxathiol-6-yl acetate (II) Geometric parameters (Å, º)

**Table:**

C1—O2	1.1936 (15)	C5—H5	0.9500
C1—O1	1.3864 (15)	C6—C7	1.3923 (16)
C1—S1	1.7709 (13)	C6—O3	1.4030 (13)
C2—C7	1.3820 (17)	C7—H7	0.9500
C2—O1	1.3835 (14)	C8—O4	1.2008 (15)
C2—C3	1.3905 (16)	C8—O3	1.3665 (14)
C3—C4	1.3908 (16)	C8—C9	1.4965 (16)
C3—S1	1.7506 (12)	C9—H9A	0.9800
C4—C5	1.3936 (16)	C9—H9B	0.9800
C4—H4	0.9500	C9—H9C	0.9800
C5—C6	1.3875 (16)		
			
O2—C1—O1	121.54 (12)	C7—C6—O3	119.12 (10)
O2—C1—S1	126.88 (10)	C2—C7—C6	115.92 (11)
O1—C1—S1	111.57 (8)	C2—C7—H7	122.0
C7—C2—O1	122.33 (10)	C6—C7—H7	122.0
C7—C2—C3	122.57 (11)	O4—C8—O3	122.31 (11)
O1—C2—C3	115.05 (10)	O4—C8—C9	127.77 (11)
C2—C3—C4	120.37 (11)	O3—C8—C9	109.92 (10)
C2—C3—S1	110.57 (9)	C8—C9—H9A	109.5
C4—C3—S1	128.97 (9)	C8—C9—H9B	109.5
C3—C4—C5	118.37 (11)	H9A—C9—H9B	109.5
C3—C4—H4	120.8	C8—C9—H9C	109.5
C5—C4—H4	120.8	H9A—C9—H9C	109.5
C6—C5—C4	119.62 (11)	H9B—C9—H9C	109.5
C6—C5—H5	120.2	C2—O1—C1	112.32 (9)
C4—C5—H5	120.2	C8—O3—C6	118.24 (9)
C5—C6—C7	123.14 (11)	C3—S1—C1	90.43 (6)
C5—C6—O3	117.42 (10)		
			
C7—C2—C3—C4	0.48 (18)	C7—C2—O1—C1	174.88 (10)
O1—C2—C3—C4	177.81 (10)	C3—C2—O1—C1	−2.46 (14)
C7—C2—C3—S1	−176.35 (9)	O2—C1—O1—C2	−177.20 (11)
O1—C2—C3—S1	0.98 (13)	S1—C1—O1—C2	2.77 (12)
C2—C3—C4—C5	−0.77 (18)	O4—C8—O3—C6	2.67 (17)
S1—C3—C4—C5	175.41 (9)	C9—C8—O3—C6	−177.05 (10)
C3—C4—C5—C6	−0.02 (17)	C5—C6—O3—C8	−111.17 (12)
C4—C5—C6—C7	1.17 (18)	C7—C6—O3—C8	75.10 (14)
C4—C5—C6—O3	−172.28 (10)	C2—C3—S1—C1	0.52 (9)
O1—C2—C7—C6	−176.54 (10)	C4—C3—S1—C1	−175.96 (12)
C3—C2—C7—C6	0.60 (17)	O2—C1—S1—C3	178.09 (12)
C5—C6—C7—C2	−1.43 (17)	O1—C1—S1—C3	−1.88 (9)
O3—C6—C7—C2	171.91 (10)		

### 2-Oxo-1,3-benzoxathiol-6-yl acetate (II) Hydrogen-bond geometry (Å, º)

**Table:**

*D*—H···*A*	*D*—H	H···*A*	*D*···*A*	*D*—H···*A*
C4—H4···O4^i^	0.95	2.35	3.2606 (15)	159
C9—H9*C*···O2^ii^	0.98	2.50	3.4030 (16)	153

Symmetry codes: (i) *x*−1, −*y*+1/2, *z*−1/2; (ii) −*x*+1, −*y*, −*z*+1.
